# A Rare Presentation of Multi-System Inflammatory Disease in Children Associated With Severe Acute Respiratory Syndrome Coronavirus 2 (SARS-CoV-2)

**DOI:** 10.7759/cureus.10892

**Published:** 2020-10-10

**Authors:** Shayan Makvandi, Omar Alibrahim, Rabheh Abdul-Aziz, Mohammad Abdul-Fattah Sallam, Megan McGreevy

**Affiliations:** 1 Department of Pediatrics, Oishei Children's Hospital/University at Buffalo, Buffalo, USA; 2 Pediatric Critical Care, Oishei Children‘s Hospital/University at Buffalo, Buffalo, USA; 3 Department of Pediatric Rheumatology, Oishei Children‘s Hospital/University at Buffalo, Buffalo, USA; 4 Pediatric Cardiology, Oishei Children‘s Hospital/University at Buffalo, Buffalo, USA

**Keywords:** multi-system inflammatory disease in children (mis-c)

## Abstract

Management of multi-system inflammatory disease in children (MIS-C) remains a challenge due to the evolving nature of the coronavirus disease 2019 (COVID-19) pandemic. This article reports a rare presentation of multi-system inflammatory disease in a previously healthy 16-month-old male who fully recovered with minimal residual cardiac insufficiency upon discharge. Our case is unique due to patient's young age, cardiac findings, and his response to our treatment protocol. A multi-disciplinary team in a tertiary center was involved with care.

## Introduction

Since April 2020, multiple reports emerged from Europe and later from New York of multi-system inflammatory disease in children (MIS-C) presenting with different clinical patterns that occur from one to six weeks following severe acute respiratory syndrome coronavirus 2 (SARS-CoV-2) infection in the pediatric age group [1-8]. Different clinical patterns were reported, including Kawasaki disease (KD)-like illness, vasoplegic shock, cardiogenic shock/myocarditis, and hyperinflammatory clinical pattern with the absence of shock and mucocutaneous involvement [2]. Cardiac involvement was variable among reports with suggested pathophysiology of cardiac edema rather than acute necrotic inflammatory myocardial injury [1]. Cardiac magnetic resonance imaging (MRI) examination supported this theory by finding little evidence of myocardial cell degeneration and necrosis [9]. The median age of affected children with MIS-C is reported to be consistently older than children affected by KD (mean: 8.5 - 10) [5, 1]. Evidence of SARS-CoV-2 infection was also variable among the reports, ranging from 69% to 100% [4-6].

## Case presentation

A previously healthy 16-month-old male presented with febrile seizure, rash, and diarrhea. Initial workup in the emergency room, including a CT scan of the head, was not conclusive. He was sent home and returned 12 hours later with 40.4 C fever, worsening upper body rash, and diarrhea. Vital signs were significant for heart rate 120, hypotensive with blood pressure 72/40, tachypneic with respiratory rate 36, and oxygen saturation of 99% while breathing ambient air. Physical exam was prominent for periorbital edema, hepatomegaly 4cm below the right costal margin, bilateral inguinal lymphadenopathy, and slightly raised non-pruritic erythematous rash concentrated on the trunk and proximal arms.

**Figure 1:**
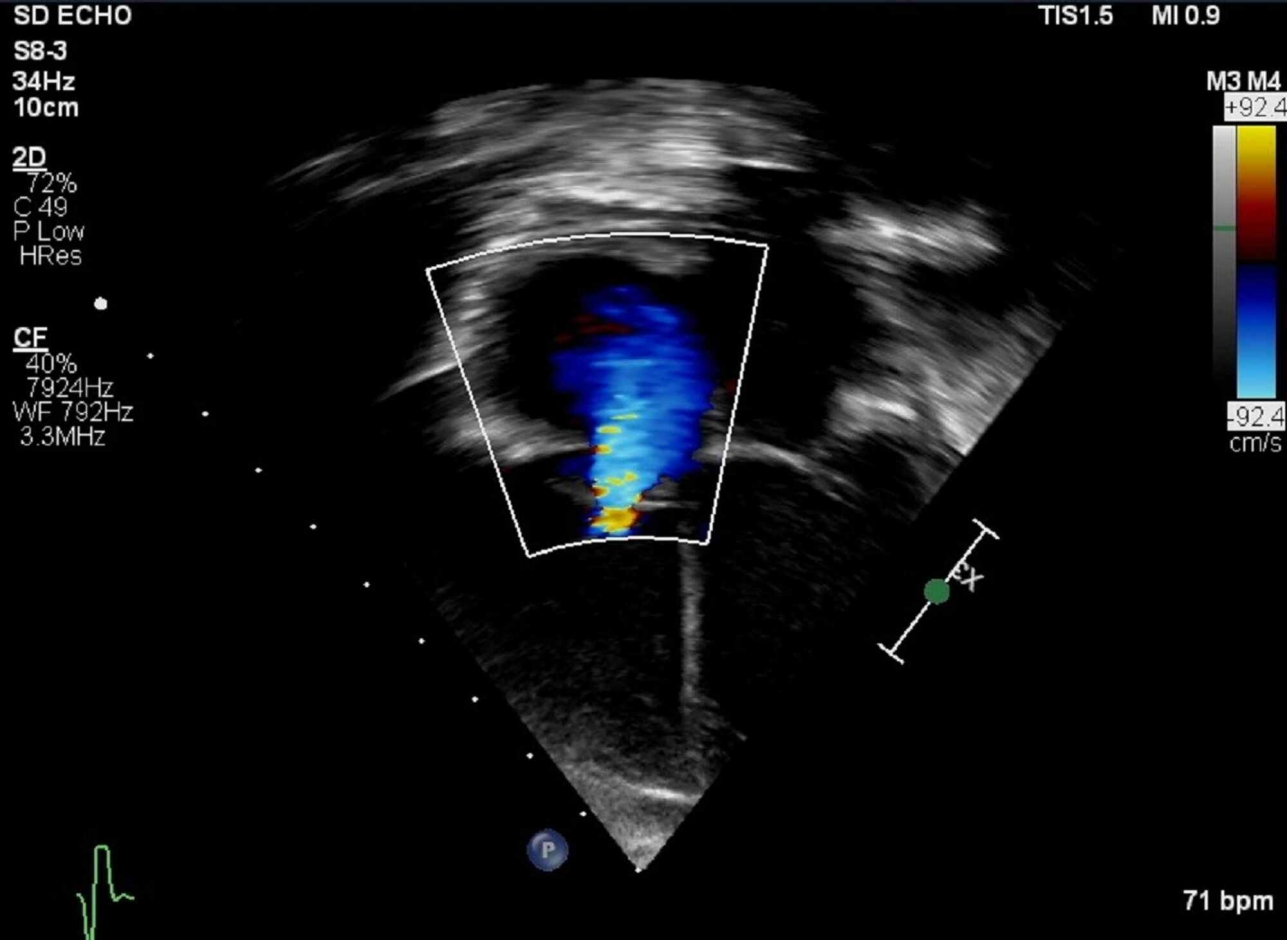
Tricuspid valve regurgitation

**Figure 2:**
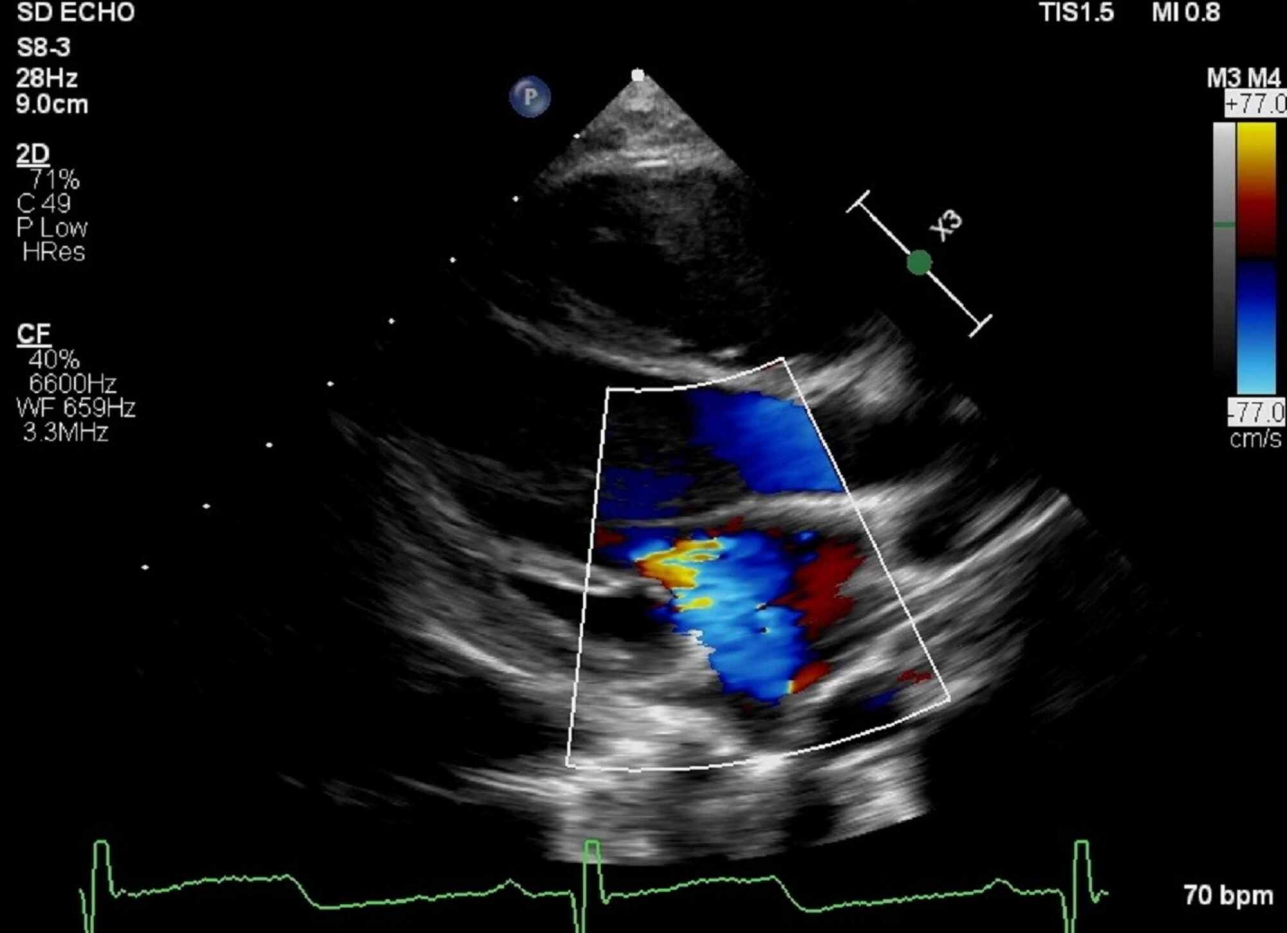
Mitral valve regurgitation

Shortly after admission, he developed distributive shock with worsening hypotension and tachycardia. The patient was intubated with concern for acute hypoxic respiratory failure and shock state. Vasopressors and inotropes were initiated for hypotension. Blood and urine cultures were obtained, and broad-spectrum antibiotics with cefepime and vancomycin were started. Patient met Centers for Disease Control and Prevention (CDC) criteria for MIS-C with fever, anemia hemoglobin 8.8 g/dL, hypoalbuminemia 2.7 g/dL, thrombocytopenia 69 x10^9/L, elevated C-reactive protein 134 mg/L, erythrocyte sedimentation rate 10mm/hr, elevated procalcitonin 33.9 ng/mL, elevated ferritin 2763 ng/mL, elevated lactic acid dehydrogenase 987 u/L, IL6 318pg/mL, IL2 4705pg/mL, IL10 38pg/mL, coagulopathy with significantly elevated D-dimer 18 mcg/mL, prolonged prothrombin time (PT) 17.9 sec, prolonged partial thromboplastin time (PTT) 39.3 sec, elevated brain natriuretic peptide (BNP) 498 pg/mL and low anti-thrombin III 39, positive COVID-19 immunoglobulin G (IgG) antibody and negative polymerase chain reaction (PCR). The rapid viral panel was negative. Serial echocardiograms showed hyperdynamic left ventricle systolic function and mild tricuspid regurgitation with no clinical significance and ultimately resolved (Figures 1-2). EKG showed nonspecific t-wave changes. Troponin was within a normal range. The patient received methylprednisolone 20mg/kg, anakinra 2mg/kg, intravenous immunoglobulin (IVIG) 2g/kg, and prophylactic Lovenox® dosing with 0.5mg/kg twice daily. The patient remained intubated on mechanical ventilation and remained on vasopressors/inotropes for three days with a maximum epinephrine dose of 0.08 mcg/kg/min and norepinephrine 0.15 mcg/kg/min. Anakinra dosage was gradually increased over time due to persistent fevers. The patient completed 12 days of anakinra with a maximum dosing of 12mg/kg/day, two doses of IVIG, and was maintained on a high dose of methylprednisone 30 mg/kg/day on the second and third days and then 8mg/kg/day with tapering for the rest of stay, which switched to oral prednisone once oral tolerance improved. The patient stayed at the hospital for a total of 15 days and was discharged on steroid taper. Laboratory data for the initial 10 days of admission are presented in Table 1.

**Table 1:** Laboratory data for initial 10 days of admission WBC - white blood cells; Hgb - hemoglobin; ESR - erythrocyte sedimentation rate; PT - prothrombin time; PTT - partial thromboplastin time; BUN - blood urea nitrogen; AST - serum aspartate aminotransferase; ALT - alanine aminotransferase; LDH - lactate dehydrogenase; BNP - brain natriuretic peptide; CRP - C-reactive protein

Date	6/6	6/7	6/8	6/9	6/10	6/11	6/12	6/13	6/15	6/18
WBC x10^9/L	7.7	5.5	8.5	7.2	12.6	12.9	12.1	15	10.1	12.2
Hgb x10^12/L	8.0	6.2	7.6	8.1	8.3	9.1	9.3	9.0	9.3	8.7
Platelets x10^9/L	69	68	73	102	202	248	306	397	382	336
Neutrophils/ 100 leukocyte	44	62	66	66	61	61	67	37	42	34
Lymphocyte/ 100 leukocyte	18	35	28	28	32	25	18	46	38	52
Monocytes/ 100 leukocyte	1	0	1	4	3	10	13	17	15	14
ESR (mm/hr)	10	22	-	11	-	21	-	18	19	12
Ferritin (ng/mL)	2763	4465	2566	2367	1402	1184	1182	774	496	238
PT (sec)	16.9	15.9	14.2	13.8	14.3	14.1	15.1	-	-	-
PTT (sec)	39.3	45.4	42.6	33.1	27.9	27.3	31.1	-	-	-
Fibrinogen	309	315	-	151	-	141	-	103	125	131
D-dimer (mcg/mL)	18.27	14.78	-	14.22	-	18.1	-	6.37	2.2	1.02
Antithrombiin III level	39	-	52	78	92	97	92	-	-	-
Sodium (mmol/L)	138	139	138	142	140	140	135	-	137	-
Potassium (mmol/L)	4.0	3.1	2.6	3.1	3.3	4.5	4.8	-	4.7	-
Chloride (mmol/L)	115	112	106	103	102	106	103	-	104	-
Carbon dioxide (mmol/L)	15	14	20	27	27	25	23	-	23	-
BUN (mg/dL)	11	9	14	10	11	13	11	-	19	-
Creatinine (mg/dL)	0.36	0.37	0.53	0.47	0.42	0.45	0.45	-	0.43	-
Calcium (mg/dL)	6.6	6.4	6.9	7.3	8.1	8.6	8.7	-	9.2	-
Phosphate (mg/dL)	1.2	2	2.5	1.7	2.3	3.3	4.5	-	-	-
Magnesium (mg/dL)	1.6	1.7	1.7	1.8	1.8	1.9	2.1	-	-	-
Lactate (mmol/L)	4	1.2	0.9	0.9	1.2	-	-	-	-	-
Bilirubin (mg/dL)	3.5	1.5	1.6	1.9	-	-	-	-	-	-
AST (unit/L)	104	83	50	55	-	-	-	-	-	-
ALT (unit/L)	85	66	36	41	-	-	-	-	-	-
LDH (unit/L)	987	679	-	456	-	602	-	556	408	309
Glucose (mg/dl)	117	127	133	130	98	83	93	-	87	-
Triglycerides (mg/dL)	292	156	-	387	-	236	-	212	-	307
BNP (pg/mL)	498	-	-	1181	1270	691	622	-	-	29
CRP (mg/L)	134.9	154.6	60.3	42.7	34.18	27.9	19.09	14.36	-	5
Procalcitonin (ng/mL)	33.9	78.7	89.5	33.29	9.45	-	1.27	-	-	-

## Discussion

Management of MIS-C remains a challenge due to the evolving nature of this pandemic. A multi-disciplinary team is required to guide care. Due to the novelty of this condition, well-established treatment guidelines are not available; therefore, newly proposed institutional guidelines were utilized [8]. Clinical improvement and inflammatory markers reduction were adopted as markers for disease improvement. Our approach in pharmacological treatment was parallel to most institutions with IVIG, glucocorticoids, and interleukins receptor antagonists (Treatment guidelines: Montgomery V, Vidwan J, Statler V, et al.: EBC Guideline: Evaluation for Multisystem Inflammatory Syndrome - Children and Management of MIS-C. Norton Children’s hospital, 2020). Other institutions used biologic modifying agents only in patients unresponsive to IVIG and corticosteroids [3, 5]. Early development of shock with cardiac dysfunction has been the hallmark of MIS-C in acutely ill patients [3]. The positive outcome of this case emphasizes the importance of early recognition of shock state, proper and judicious fluid resuscitation, the early establishment of invasive monitoring, intubation, mechanical ventilation, and appropriate initiation of inotropes and vasopressors. Almost half of the patients presenting with MIS-C show some degree of ventricular dysfunction, pericardial effusion, or coronary aneurism. The rate of cardiac dysfunction without coronary involvement is much higher in MIS-C compared to KD [9]. Notably, patients with MIS-C have a higher prevalence of shock (76%) and cardiac dysfunction compared to Kawasaki disease (3%) [3]. Our patient showed noticeable bilateral atrioventricular (AV) valves regurgitation, which is a new finding compared to documented cases [10]. Persistence of cardiac dysfunction has been noticed in many cases on discharge [3]. This requires a close follow up of recovered patients with serial ECHOs. Concomitant cytokine storm in some patients with MIS-C makes it challenging to treat patients with only one clinical approach. There is not enough data for early prophylactic anticoagulation for the pediatrics population with MIS-C. In our patient with multiorgan failure and significant coagulation derangement, anticoagulation was justified. In some institutions, aspirin is used for initial anticoagulation, and low molecular weight heparin (LMWH) is only used in patients with noticeable elevation in D-dimer [3]. Further studies are warranted to investigate the pathophysiological mechanism of this post-infectious immune-mediated disease.

## Conclusions

The unique about our case is the young age of the patient, unique cardiac involvement with valvar regurgitation, patient’s initial presentation with seizure, and response to our treatment protocol. Up to date, there is no approved treatment protocol for similar critical cases. It is important to share our experience with providers taking care of these newly evolving critical cases
